# Functional redundancy and sensitivity of fish assemblages in European rivers, lakes and estuarine ecosystems

**DOI:** 10.1038/s41598-017-17975-x

**Published:** 2017-12-14

**Authors:** Nils Teichert, Mario Lepage, Alban Sagouis, Angel Borja, Guillem Chust, Maria Teresa Ferreira, Stéphanie Pasquaud, Rafaela Schinegger, Pedro Segurado, Christine Argillier

**Affiliations:** 1 Irstea, UR EABX, Equipe FEE, av. de Verdun, F-33612 Cestas, France; 2 Irstea, UR RECOVER, Equipe FRESHCO, 3275 Route de Cézanne, CS 40061, 13182 Aix en Provence, France; 3 AZTI, Marine Research Division, Herrera Kaia Portualdea s/n, 20110 Pasaia, Spain; 4 Centro de Estudos Florestais, Instituto Superior de Agronomia, Universidade de Lisboa, Tapada da Ajuda, 1349-017 Lisbon, Portugal; 5 MARE- Marine and Environmental Sciences Centre, Faculdade de Ciências, Universidade de Lisboa, Campo Grande, 1749-016 Lisboa Portugal; 6 Institute of Hydrobiology and Aquatic Ecosystem Management, Department of Water, Atmosphere and Environment, BOKU, University of Natural Resources and Life Sciences, Vienna, Austria

## Abstract

The impact of species loss on ecosystems functioning depends on the amount of trait similarity between species, i.e. functional redundancy, but it is also influenced by the order in which species are lost. Here we investigated redundancy and sensitivity patterns across fish assemblages in lakes, rivers and estuaries. Several scenarios of species extinction were simulated to determine whether the loss of vulnerable species (with high propensity of extinction when facing threats) causes a greater functional alteration than random extinction. Our results indicate that the functional redundancy tended to increase with species richness in lakes and rivers, but not in estuaries. We demonstrated that i) in the three systems, some combinations of functional traits are supported by non-redundant species, ii) rare species in rivers and estuaries support singular functions not shared by dominant species, iii) the loss of vulnerable species can induce greater functional alteration in rivers than in lakes and estuaries. Overall, the functional structure of fish assemblages in rivers is weakly buffered against species extinction because vulnerable species support singular functions. More specifically, a hotspot of functional sensitivity was highlighted in the Iberian Peninsula, which emphasizes the usefulness of quantitative criteria to determine conservation priorities.

## Introduction

The global ecological footprint of human activities entailed a rapid decline of biodiversity over the past decades^1^, which affects functions and services delivered by ecosystems^2^. The growing concern about the acceleration of species’ extinction rate has led to increasing conservation science research initiatives to assess the role of biodiversity in the resilience and stability of ecosystems^3^. According to the insurance hypothesis of biodiversity, the maintenance of high diversity and redundancy in functional traits contributes to increase the stability of biological assemblages and their associated ecological processes^4^. The variability in responses to disturbances among species sharing similar functions ensures ecosystem recovery after disturbance by compensating the loss of functionally redundant species^5,6^. Therefore, the influence of species loss on ecosystem functioning is expected to be greater in less redundant assemblages, especially if singular traits are supported by vulnerable or endangered species^7,8^. The assessment of functional sensitivity to species loss reflecting the tendency of the community to be affected by stressors is thus a crucial issue in environmental management to prevent alteration of ecosystem functioning subjected to human-induced disturbance^9^.

The impact of species loss on functional diversity depends on the amount of redundancy, but it is also influenced by the order in which species are lost^10^. Species respond in different ways to environmental disturbances, so that extinction or population decline are not necessarily random processes^11^. The species extinction risk is consistently related to intrinsic components driving specific response to disturbance, e.g. life-history traits, habitat requirements, population size etc., but also depends on extrinsic factors, such as the intensity of threats^12,13^. The combination of these factors influences the order of species’ extinction within an assemblage, and consequently leads to a non-random pattern of functional diversity loss^6,14^. Therefore, the functional sensitivity of assemblage will be higher if vulnerable species support non-redundant functions. Such cases were reported for diverse fauna^15^, including freshwater fish^8^, where the loss of few threatened or endangered species can cause a great decline in functional diversity.

Aquatic ecosystems largely contribute to maintaining overall environmental health and provide goods and services for human populations, such as aquatic resources for food or nutrient regulation^16^. Key ecosystem services are also connected to the hydrological cycle in the river basin, for example water purification, water retention and climate regulation^17^. However, these ecosystems are globally threatened by human activities that cause habitat degradation, connectivity loss, water pollution, resource overexploitation, or introduction of alien species, which result in a rapid aquatic biodiversity decline^18^. About 2251 (41%) of the 5435 animal species present in the 2000 Red list of the International Union for Conservation of Nature (IUCN) live in marine and inland water environments^19^. Effective management and conservation strategies are thus required to maintain high biodiversity levels to ensure the long-term sustainability of ecosystem functioning, resilience, and delivered services^20^. The decline of fish species’ diversity can produce drastic changes in ecosystem functioning because of their implication in food web dynamics, nutrient flows, or redistribution of bottom sediment^21^. For several decades, monitoring programs conducted in continental aquatic ecosystems took advantage of the key role of fish assemblages to assess ecosystem health on the basis of ecological indicators^22,23^. Although these indicators are relevant management tools, they do not provide quantitative information to evaluate the potential impact of species loss on ecosystem functioning. To this aim, the assessment of species functional role in ecosystems is essential to guide conservation efforts toward the preservation of the most original species^7,24^ and sensitive assemblages composed of poorly redundant species^9^. Moreover, the development of effective management strategies should consider the specificity of the continental aquatic ecosystems, that are distributed from the source to the estuaries, to conduct the most relevant conservation actions^25^. Indeed, fish assemblages occupying lake, river and estuarine ecosystems can display different functional richness and redundancy patterns, which should be taken into account to determine their functional sensitivity to species loss.

Hence, in this contribution, we investigated the consequence of species loss on functional richness in lakes, rivers, and estuaries, with the aim of assessing functional sensitivity of fish assemblages across the three main continental aquatic systems. Particularly, we investigated the shape of the relationship between species and functional richness to compare redundancy patterns^10^ between aquatic systems in France, Spain, and Portugal. This relationship is expected to be linear when all species of an assemblage support singular functions, meaning that the loss of any species will produce an important and equivalent decline in functional richness^26^. On the contrary, functionally redundant assemblages will display curvilinear relationships, i.e. saturation trends, as some functional traits are shared by multiple species. This property was used to assess the functional redundancy of fish assemblages and, by extension, the impact of species loss on ecosystem functioning. More specifically, we attempted to address three main issues: 1) Do fish assemblages have different functional sensitivities to species loss in the three aquatic systems? 2) Does the functional redundancy increase with taxonomic richness in these systems, thus promoting resilience against the loss of ecological functions? and 3) Does the loss of vulnerable species cause a greater functional alteration than random extinction? To answer these questions, we conducted several scenarios of species extinction within assemblages where species are removed sequentially depending on ecological features related to species sensitivity to environmental disturbances.

## Methods

### Available data

We used data collected within monitoring programs related to the EU Water Framework Directive (WFD; 2000/60/EC) to obtain species lists and estimates of fish abundance in 49 estuaries, 302 lakes, and 869 river reaches distributed throughout the south-western Europe, i.e. France, Spain and Portugal. The study area covered two main biogeographical regions separated by the Pyrenees Mountains, i.e. the Peri-Mediterranean region thereafter referred as Iberian region and the Danubian region^27^. The river sites were assigned to four categories of fish assemblages, i.e. headwater streams (HWS), medium gradient rivers (MGR), lowland rivers (LLR), and Mediterranean streams (MES), that differs in their fish community and environmental characteristics^28^. Estuaries and lakes were assigned to two size categories, i.e. small and large, because habitat availability is a major factor affecting fish assemblages in these systems. The size thresholds were set to 0.68 km² and 25 km², respectively for lakes and estuaries, according to system size and previous knowledge^29,30^.

The WFD requirements ensure the availability of relatively homogeneous and comparable fish datasets for each aquatic system in terms of standardization of sampling efforts and fishing techniques^23^. For estuarine systems, fish abundances were estimated on the basis of beam trawl surveys during autumn and spring periods. The protocol consists of performing several hauls distributed across the whole salinity gradient following standardized requirements of the country^31–33^. The number of hauls is defined according to the system size to ensure the sampling representativeness. Abundances of all taxa were standardized by dividing the number of individuals by the sampled surface (expressed in number of individuals per 1000 m²). For lakes, fish data were obtained by application of the Norden gillnet standardised protocol^34^. Benthic multi-mesh gillnets were set in different depth strata during the summer period and the sampling effort depended on lake depth and area. Nets were set before dusk and lifted after dawn to cover the activity peaks of all the fish species. Fish abundance were standardised by computing catch per unit effort (CPUE, number of individuals caught by m² by night). For rivers, fish data were extracted from an extensive database^35^ containing fish surveys conducted by several academic institutions and environmental agencies across Europe. Sites were sampled by electrofishing (wading) during low flow periods using European standards^36^. Abundances are expressed in number of individuals per m². To minimise the risk of false absences, we included only sites where fished areas were greater than 100 m² with more than 50 individuals caught. For the three aquatic systems, fish assemblages were determined in terms of species occurrence and abundance by pooling the available samplings of each locality.

The analyses were conducted on 271 species, which included 24 non-native species. These species were more common in lakes and rivers, with 12 exotic and 12 translocated species, than in estuaries where only one exotic and four translocated species were recorded. Although non-native species contribute to increase the functional richness^37^, their exclusion would alter the current view of functional structure of fish assemblages, because introduction process is occurring since the 16^th^ century in this region. Therefore, no detailed focus was led on non-native species, but taking out those species would provide additional insights to reconstruct the impact of introduction history in further investigations.

### Fish functional traits

The functional niches of fish are described based on five complementary traits, which are commonly used in studies examining functional diversity in fish assemblages^9,38–41^: fish size, vertical position, spawning habitat, trophic group, and swimming mode. They reflect different ecological functions of species in aquatic ecosystems, focusing on key elements determining species habitat preference and position in the food web (see Supplementary Information for further details and Supplementary Dataset for data availability). We used coarse categorical traits, as the detail level of ecological information is highly heterogeneous between species and did not allow accounting for possible ontogenetic shifts in functional traits. Fish body size corresponds to the maximum total length reported in literature and was coded using six ordered categories: 0–8, 8.1–15, 15.1–30, 30.1–50, 50.1–80, and > 80.1 cm. Position in the water column was expressed in two categories, i.e. benthic and non-benthic, characterizing the habitat usually used by fish for living and feeding. Species were assigned in seven trophic categories according to the dominant food item in their diet: piscivorous, omnivorous, planktivorous, insectivorous, herbivorous, detritivorous, and parasitic. Spawning habitat denotes the preference of species for specific reproductive conditions, and was coded using six categories: lithophilic, pelagophilic, phytophilic, polyphilic, nest builder and internal brooder. Swimming mode reflects the body-shape and swimming factor commonly used to describe locomotive performances of fish. It was coded using eight categories: carangiform, sub-carangiform, diodontiform, anguilliform, labriform, balistiform, amiiform and rajiform. Information about the five functional traits were obtained from FishBase^42^ and joint researches on fish assemblages in rivers^43^ and lakes^44^.

### Measure of functional richness

The functional diversity of fish assemblages was described using a dendrogram-based measure, which reflects the richness component of functional diversity^45^. This index captures the extent of complementarity among species of a local assemblage by measuring the total branch length of a dendrogram, summarizing the functional distances between species in the trait space^46^. The index increases when functionally dissimilar species are added to the assemblage but it cannot increase if a species is removed^47^. This measure of functional richness was preferred to indices derived from a multidimensional functional space^48^, such as the convex hull volume, which requires that the number of species is greater than the number of trait dimensions. Conversely, dendrogram-based measures can be achieved regardless of the number of species in the assemblages. This property is essential, as the extinction scenarios proposed in the present study are based on species subtraction, where the functional index must be calculated until species richness reaches one species only. We calculated a distance matrix between all pairs of species using the Gower dissimilarity index, which handles categorical and ordered variables^49^. An Unweighted Pair Group Method with Arithmetic Mean (UPGMA) clustering algorithm was then applied to produce a functional dendrogram, as it provided the highest cophenetic correlation coefficient with the initial distance matrix (c = 0.74) compared to other clustering methods^50^.

### Species richness and functional richness

The relationships between species richness and functional richness were examined in rivers, lakes and estuaries using multiple linear regressions. We tested for the saturation effect of the relationship by integrating a quadratic term of species richness as predictor variable. The assumptions of normality, homoscedasticity and independence of the residuals were graphically verified. A significant positive quadratic term indicates an increasing trend in functional redundancy for species-rich assemblages, whereas a linear relationship reflects a proportional contribution of supplementary species to functional richness. This analysis aimed to determine if higher species richness within assemblages is associated to higher values of functional redundancy for the three aquatic systems.

### Functional redundancy and extinction scenarios

**Figure 1:**
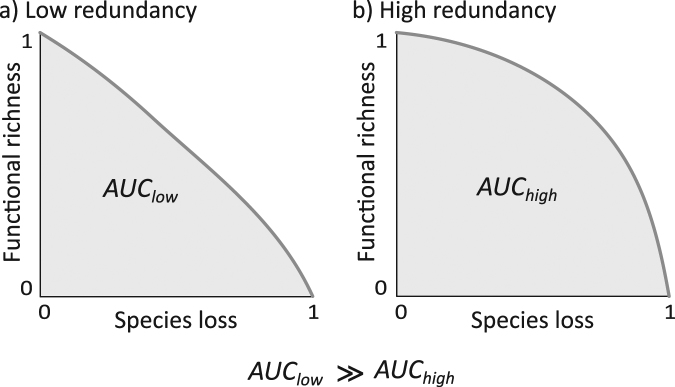
Illustrative example of extinction scenarios for two fish assemblages composed of species with (**a**) low redundant functional traits, and (**b**) highly redundant functional traits. The effect of species loss on functional richness is assessed by calculating the Area Under the Curve (AUC) defined by the residual proportion of functional richness against the proportion of species lost. Lower AUC values denote high functional sensitivity to species loss due to the limited redundancy of fish assemblages or the early loss of singular species along the extinction trajectory. In contrast, higher AUC values indicate greater functional redundancy and trajectories where redundant species tend to disappear first.

The functional redundancy and the impact of species loss on functional richness were assessed by simulating extinction scenarios under several hypotheses to investigate the relationship between species and functional richness. The simulation removed species sequentially and re-calculated the functional richness after each species’ subtraction, until functional richness was equal to zero, i.e. only one species was remaining. The impact of species loss on functional richness was then assessed by calculating the Area Under the Curve (AUC) defined by the residual proportion of functional richness against the proportion of species lost^51^ (Fig. 1). Lower AUC values denote high functional sensitivity to species loss due to the limited redundancy of fish assemblages or the early loss of singular species along the extinction trajectory. On the contrary, higher AUC values indicate greater functional redundancy and trajectories where redundant species tend to disappear first.

We simulated six extinction scenarios that differed in their trajectories: (i) a random scenario, (ii) a best-case scenario, (iii) a worst-case scenario, (iv) an abundance-based scenario, (v) a trait-based scenario, and (vi) an IUCN-based scenario. For the random scenario, species were removed sequentially assuming an equal probability of extinction between species. We simulated 999 random trajectories for each assemblage. Then, the AUC values derived from these 999 random trajectories were averaged to obtain an index of functional redundancy, which reflects the overall compensatory potential of fish assemblages. The best- and worst-case scenarios reflect extreme trajectories where the order of species extinction minimises or maximises the loss of functional richness at each species lost. At each step, the species to remove were determined by calculating the functional loss associated to all species still comprised in the assemblage and then selecting those that produced the smallest (best-case) or largest (worst-case) decline in functional richness. In the abundance-based scenario, species extinction occurs in inverse order of the abundance within an assemblage, reflecting a plausible scenario where rare species disappear first. Species were ranked according to their relative abundances in assemblages using the fish surveys of each sampling site. In the trait-based scenario, the extinction order was defined by the intrinsic vulnerability score (ranging from 0 to 100) proposed by Cheung, *et al*.^52^ and available on FishBase^42^. This composite score is calculated on the basis of life-history traits and ecological attributes that influence the resilience abilities of local populations^53^. Contrary to the IUCN assessment criteria, the scoring process does not consider the abundance and distribution range of species but reflects their intrinsic recovery abilities when facing threats, which is relevant in large-scale studies^54^. Finally, the IUCN-based scenario consisted of removing clusters of species following a decreasing order of vulnerability derived from the categories of the IUCN Red List^19^, i.e. from species listed as ‘critically endangered’ to those listed as ‘least concern’. Those categories reflect a risk of extinction for species essentially due to anthropogenic disturbances, such as pollution, habitat and connectivity alteration, introduction of alien species or overexploitation of resources^55^. Therefore, this assessment provides useful quantitative criteria to forecast the impact of extinction of threatened species on functional richness, contributing thus to determine sensitive areas where the species loss can induce a sharp alteration of ecosystem functioning^8^. For analysis, the species for which the IUCN assessment was not completed were included in the ‘least concern’ category, so that they were removed in latter position.

### Comparison with random extinctions

The AUC values obtained from the directional scenarios (abundance-, trait- and IUCN-based scenarios) were compared to those calculated from random trajectories to determine whether the functional richness was more strongly affected by these plausible scenarios than by random extinctions. To this end, we calculated standardised effect sizes (*SES*) according to the formula,

$$SES=(Obs-Mea{n}_{r})/s{d}_{r}$$

where *Obs* was the AUC value of the directional scenario, *Mean*_*r*_ and *sd*_*r*_ were respectively the mean and standard deviation of AUC values obtained from the 999 random trajectories. Negative SES AUC values indicated assemblages where functional richness was affected more strongly by the impact of directional scenarios than random expectation. Note that the IUCN-based scenarios were compared to random trajectories where species were removed by cluster of the same number than species listed in IUCN categories. For each aquatic system, we tested if the SES AUC values of directional scenarios were significantly lower than zero, based on unilateral Wilcoxon rank tests. Similar tests were also conducted within aquatic systems by using fish assemblages included in the two biogeographical regions, i.e. Iberian and Danubian, the river categories, i.e. HWS, MGR, LLR, and MES, and the size categories, i.e. small and large, for lakes and estuaries.

### Statistical considerations

The statistical analyses were performed using the R software^56^ supplemented by the package ‘vegan’^57^ for the computation of dendrogram-based measures. Accounting that the data do not meet the assumptions of normality and variance homogeneity among groups, non-parametric tests were used to determine the significance of changes between aquatic system types. Kruskal–Wallis non-parametric tests were used to compare indices between multiple systems and *post-hoc* pairwise comparisons were conducted based on Wilcoxon tests. For *post-hoc* tests, the alpha level was adjusted using the Bonferroni correction to avoid an inflated Type I error rate. The association between species richness and functional redundancy was assessed using the Spearman correlation test. For all tests, the significance threshold was set at α = 0.05. The maps were built in the R environment using the packages ‘sp’^58^ and ‘rgeos’^59^.

## Results

### Species richness, functional richness and redundancy

**Figure 2:**
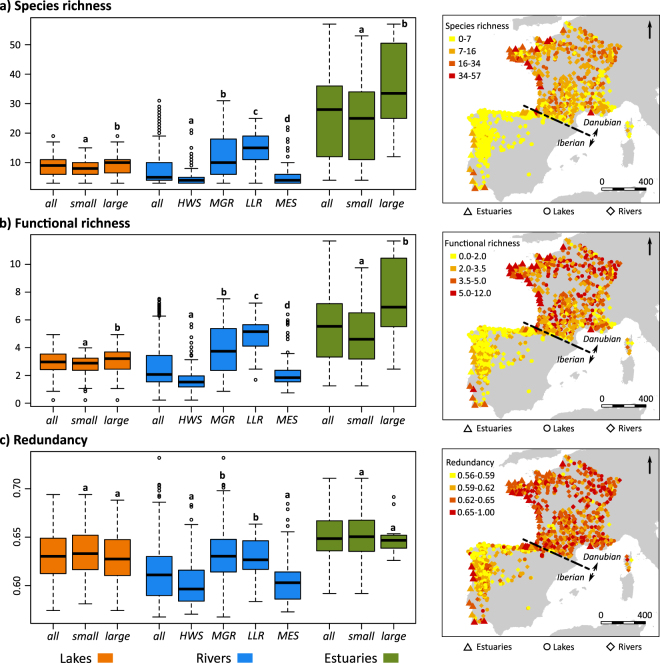
Species richness (**a**), functional richness (**b**) and functional redundancy (**c**) of fish communities across lakes, rivers and estuaries in France, Spain and Portugal. Boxplots represent the general trends for each aquatic system (*all*), as well as trends for the system categories, i.e. *small* and *large* lakes, *small* and *large* estuaries, headwater streams (*HWS*), medium gradient rivers (*MGR*), lowland rivers (*LLR*), and Mediterranean streams (*MES*). For each aquatic system, letters indicate categories that were not significantly different after a post-hoc Kruskal–Wallis test (*P*-value ≥ 0.05). The maps display the geographical distribution of indices values. Functional diversity was calculated from a combination of five traits, i.e. fish size, vertical position, trophic group, spawning habitat, and swimming mode. The functional redundancy of each assemblage was assessed from the average area under the curve of 999 random trajectories of species extinctions. The maps were built using the libraries ‘sp’ and ‘rgeos’ available in R software (www.r-project.org) and the shapefile of the European coastline was provided by the European Environment Agency and is freely available at https://www.eea.europa.eu/data-and-maps.

We described the functional traits of 271 species from 63 families occurring in the investigated aquatic systems. Species richness was overall higher among fish assemblages of estuaries (mean = 26.7; range = 4–57) than those of rivers (mean = 7.9; range = 3–31) and lakes (mean = 8.9; range = 3–19; Kruskal-Wallis test, n = 1220, *P* < 0.001). For the three aquatic systems, the number of species was significantly higher in the Danubian region than in the Iberian region (Table 1). Additionally, the species richness varied significantly within categories of each aquatic system, with higher values reported for large lakes and estuaries, as well as MGR and LLR (Fig. 2). Overall, functional richness followed similar trend as species richness in term of geographical patterns (Table 1) and differences between (Kruskal-Wallis test, n = 1220, *P* < 0.001) and within aquatic systems (Fig. 2).

**Table 1:** Mean values of species richness, functional richness and redundancy of fish communities across lakes, rivers and estuaries in the two main biogeographical regions covered by the study area, i.e. Danubian region and Iberian region.

	Lakes			Rivers			Estuaries		
	Danubian	Iberian	*P*-value	Danubian	Iberian	*P*-value	Danubian	Iberian	*P*-value
Species richness	9.76	4.47	**<0.001**	12.24	4.29	**<0.001**	36.61	13.43	**<0.001**
Functional richness	3.16	1.88	**<0.001**	3.88	1.72	**<0.001**	7.41	3.22	**<0.001**
Redundancy	0.64	0.60	**<0.001**	0.63	0.60	**<0.001**	0.65	0.65	0.592

*P*-values indicate the significance of changes between regions for each aquatic system using Kruskal–Wallis non-parametric tests. Values in bold indicate *P*-value < 0.05.

**Figure 3:**
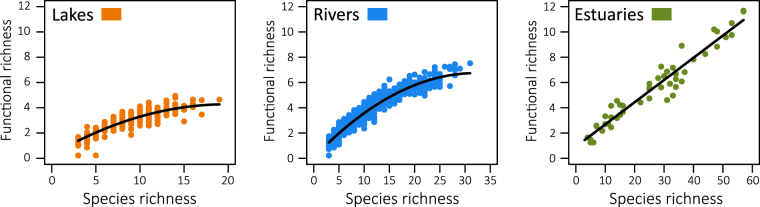
Relationship between functional richness and species richness in fish assemblages of lakes, rivers and estuaries. The saturation effects for lakes and rivers indicate an increasing trend in functional redundancy for species-rich assemblages. Note that the x-axis scale differs between aquatic systems. Functional metrics were calculated from a combination of five traits, i.e. fish size, vertical position, trophic group, spawning habitat, and swimming mode.

Functional richness was closely related to species richness of fish assemblages in lakes, rivers and estuaries (Fig. 3). The quadratic terms of species richness integrated in regression models were significant for lakes (*F*-test, n = 302, *P* < 0.001) and rivers (*F*-test, n = 869, *P* < 0.001), which indicates a saturation effect in these relationships (lakes, r² = 0.77; rivers, r² = 0.94). Conversely, the effect of the quadratic term was not significant for estuaries (*F*-test, n = 49, *P* = 0.42), revealing that the linear model was a more parsimonious way to describe the relationship (r² = 0.93). This pattern was confirmed by the functional redundancy calculated from the random trajectory, which was positively correlated with the species richness in lakes (Spearman correlation test, n = 302, *rho* = 0.60, *P* < 0.001) and rivers (Spearman correlation test, n = 869, *rho* = 0.72, *P* < 0.001), but it was not influenced by the number of species in estuaries (Spearman correlation test, n = 49, *rho* = 0.12, *P* = 0.402). Overall, fish assemblages in estuaries showed a higher level of redundancy (mean = 0.65, range = 0.59–0.71) than in lakes (mean = 0.63, range = 0.57–0.69) and in rivers (mean = 0.61, range = 0.56–0.73; Kruskal-Wallis test, n = 1220, *P* < 0.001). The functional redundancy of fish assemblages was higher in lakes and in rivers located in the Danubian region, whereas no difference was identified between biogeographical regions for estuaries (Table 1). Despite changes in species and functional richness, the redundancy was not significantly affected by the size categories of lakes and estuaries (Fig. 2). Conversely, fish assemblages of MGR and LLR displayed higher level of functional redundancy than HWS and MES.

### Scenarios of species extinction

The AUC values calculated from the best-case scenarios were significantly correlated with the number of species for lakes (Spearman correlation test, n = 302, *rho* = 0.63, *P* < 0.001) and rivers (Spearman correlation test, n = 869, *rho* = 0.59, *P* < 0.001), but not for estuaries (Spearman correlation test, n = 49, *rho* = 0.12, *P* = 0.403), which supports the previous results. Conversely, the AUC values of worst-case scenarios were uncorrelated with species richness for the three aquatic systems (Spearman correlation tests, lakes, n = 302, *rho* = −0.09, *P* = 0.103; rivers, n = 869, *rho* = 0.03, *P* = 0.356; estuaries, n = 49, *rho* = 0.09, *P* = 0.517). This result indicates that some functional traits are supported by non-redundant species, so that their loss among first positions induces large impact on functional richness, whatever the number of species in assemblages.

**Figure 4:**
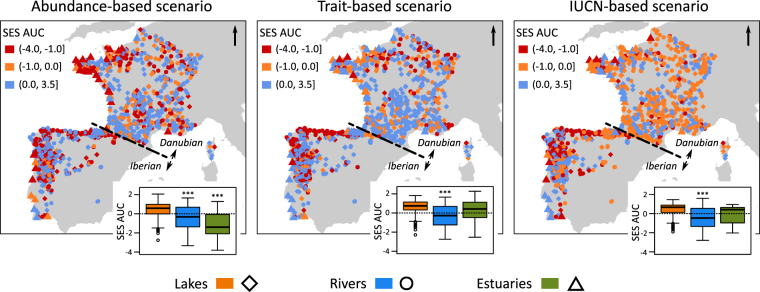
Regional map of Standardised Effect Size of Area Under the Curve (SES AUC) calculated from the abundance-based scenario (left map), the trait-based scenario (middle map) and the IUCN-based scenario (right map) for fish assemblages of lakes, rivers, and estuaries. Negative SES AUC values indicate communities in which the functional diversity is more impacted by directional scenarios compared to random trajectories of species extinction. For each scenario, the boxplots indicate the general trend for the three aquatic systems. The asterisks designate the aquatic systems in which SES values were significantly lesser than zero based on unilateral Wilcoxon rank tests (*P*-value < 0.001). The maps were built using the libraries ‘sp’ and ‘rgeos’ available in R software (www.r-project.org) and the shapefile of the European coastline was provided by the European Environment Agency and is freely available at https://www.eea.europa.eu/data-and-maps.

The SES AUC obtained from the abundance-based scenarios were significantly lower than zero for the rivers and estuaries (Fig. 4), as well as their sub-categories (Table 2), which reflects a substantial contribution of non-abundant species to the functional richness of these systems. However, note that the functional alteration caused by the loss of rare species was higher for estuaries located in the Danubian region that in the Iberian region (Table 2 and Supplementary Table S1). For this scenario, the SES AUC values did not differed significantly between the categories within the three aquatic systems (Supplementary Table S2). Both trait-based and IUCN-based scenarios simulated from the river assemblages caused greater functional alterations than random patterns of species extinction (Table 2), whereas the SES AUC were not significantly lower than zero for lake and estuarine assemblages (Fig. 4). According to these two scenarios, fish assemblages of rivers located in the Iberian region, especially the north-western Iberian Peninsula (Fig. 4), tended to be more affected by the loss of vulnerable species (Supplementary Table S1). Moreover, SES AUC values obtained from the trait-based scenario were overall higher for LLR and MES, whereas MGR tended to be less impacted under the IUCN-based scenario (Table 2, Supplementary Tables S2 and S3).

**Table 2:** Impact of directional scenarios of species extinction on the functional richness within fish assemblages of lakes, rivers, and estuaries.

System type	Region/categories	Number of sites	Abundance-based scenario		Trait-based scenario		IUCN-based scenario	
			SES AUC	*P-*value	SES AUC	*P-*value	SES AUC	*P-*value
Lakes	all	302	0.40	>0.999	0.65	>0.999	0.40	>0.999
	Danubian	255	0.47	>0.999	0.71	>0.999	0.21	>0.999
	Iberian	47	0.00	0.448	0.33	0.995	0.06	0.702
	small	99	0.40	>0.999	0.64	>0.999	0.44	>0.999
	large	203	0.40	>0.999	0.66	>0.999	0.38	>0.999
Rivers	all	869	−0.40	**<0.001**	−0.31	**<0.001**	−0.42	**<0.001**
	Danubian	394	−0.46	**<0.001**	−0.16	**<0.001**	−0.16	**<0.001**
	Iberian	475	−0.35	**<0.001**	−0.43	**<0.001**	−0.47	**<0.001**
	HWS	240	−0.31	**0.001**	−0.11	0.482	−0.60	**<0.001**
	MGR	305	−0.50	**<0.001**	−0.16	**0.032**	−0.18	**0.012**
	LLR	41	−0.51	**0.008**	−0.73	**<0.001**	−0.42	**0.003**
	MES	283	−0.36	**<0.001**	−0.57	**<0.001**	−0.49	**<0.001**
Estuaries	all	49	−1.22	**<0.001**	0.30	0.983	−0.14	0.295
	Danubian	28	−1.87	**<0.001**	0.01	0.566	−0.08	0.374
	Iberian	21	−0.35	0.064	0.69	0.997	−0.16	0.294
	small	37	−1.16	**<0.001**	0.24	0.934	−0.22	0.187
	large	12	−1.39	**0.031**	0.49	0.935	0.03	0.661

For each aquatic system and its associated categories and regions, the mean values of the Standardised Effect Size of Area Under the Curve (SES AUC) were calculated from the abundance-based, the trait-based and the IUCN-based scenarios. Negative SES AUC values indicate assemblages in which the functional diversity is more impacted by directional scenarios compared to random trajectories of species extinction. *P*-values indicate if the SES AUC values of directional scenarios were significantly lower than zero, based on unilateral Wilcoxon rank tests. Values in bold indicate *P*-value < 0.05. For each aquatic system, the number of sites is reported for the total set (all), along with for their associated biogeographical region (Danubian and Iberian) and categories (small and large; headwater streams - HWS, medium gradient rivers - MGR, lowland rivers - LLR, and Mediterranean streams - MES).

## Discussion

Species extinction is a crucial concern in conservation science, especially regarding the consequences of biodiversity loss on ecosystem stability, resilience and functioning^60^. Most previous studies reported a positive relationship between species richness and functional diversity in natural assemblages, including terrestrial^61,62^ and aquatic environments^40^, which suggests that species extinction is often associated with a severe decline in ecological functions^10^. We also found a strong positive link between species- and functional richness among fish assemblages of the three continental water systems. However, substantial differences were underlined by our analysis regarding the shape of the functional-species relationship, as well as the assemblage sensitivity to species loss.

Overall, our results indicate that fish assemblages in lakes and rivers can be more sensitive to species loss than estuarine assemblages because of their lower functional redundancy. In comparison to other regions of the globe, freshwater fish assemblages in Europe are relatively species-poor due to the intensity of the last glacial period^27^. This reduced number of species is associated with a low level of functional redundancy, which indicates that co-existing species tend to express different combinations of ecological traits, likely in response of resource partitioning process^63^. Indeed, niche partitioning was previously reported a primary factor for driving the functional structure and redundancy of native freshwater fish communities^64^. In this context, the random loss of one species is likely to result in a sharp decrease of functional richness. This process is strengthened in some categories of river, such as HWS and MES, where the very species-poor assemblages are not functionally buffered against species extinction. By contrast, the species and functional richness of estuarine assemblages are commonly higher than those of rivers and lakes, because of higher heterogeneity in environmental conditions that promote a species turnover^65^. Moreover, the estuarine environment imposes huge physiological constraints that contribute to increasing functional redundancy^66,67^, so that multiple species distributed along estuaries share similar functional attributes^68^. This broad comparison of ecosystem types supports the statement that species-poor ecosystems should require more management efforts to prevent the loss of ecological function, due to their low functional redundancy as it has been reported in marine fish^9^ and birds^62^ communities. Nevertheless, Parravicini, *et al*.^9^ demonstrated that high species richness alone cannot ensure buffer against species extinction because some functions are usually supported by unique species, even in extremely rich communities.

Our findings indicate that the increase of functional redundancy with species richness is not consistent among the three continental aquatic ecosystems. Although fairly positive for lakes and rivers, the relationship was no significant for estuaries, indicating that species-rich assemblages are not necessarily more redundant. For example, even with low species number, the functional redundancy of small estuaries was not significantly lower than that of larges estuaries. Likely, the environmental heterogeneity and availability of different habitat types in large estuaries provides favourable conditions for more species that can occupy diversified functional niches^69,70^. A similar pattern has been reported in coral reef fish, stressing that singular species and functions commonly occur even in species-rich assemblages of marine ecosystems^39,71^. By contrast, the relationship between functional- and species richness displayed an asymptotic trend for lake- and river assemblages. This indicates that functional richness increases at low species richness, but the increase rate declines by adding supplemental species because they tend to share traits already represented in the assemblage^26^. This pattern can be related to the existence of primordial functions that constitute the basic ecological core of assemblages, so that they are found in priority and become rapidly redundant^39^. Therefore, in lakes and rivers, higher taxonomic richness can contribute to promote assemblage resilience against the loss of ecological functions^72^.

Although random extinction is a standard assumption to provide a conservative estimate of assemblages sensitivity through species redundancy, extinction scenarios mediated by specific ecological features can affect the rate of functional trait loss^8,10,73^. The diversity of species responses to disturbances plays an important role for determining the compensatory potential as well as ecosystem resilience^5^. Indeed, a highly redundant function can be lost if all species that share this function are sensitive to the same threat^3^. Therefore, approaches that investigate the variability of responses to disturbances among similar performing species in terms of ecosystem function can provide more integrative assessment of ecosystem resilience^74^. However, such approach requires quantifying response diversity within functional groups, which can be problematic when the number of species in assemblages is low. Here, we approximated the species sensitivity to environmental disturbance using contextual and biological features (local abundance, demographic traits, and IUCN status) that influence species propensity to persist under anthropogenic stress, and therefore the order of species extinction. Beyond species local abundance, we used the IUCN status and the intrinsic vulnerability score of species that provide complementary indices. Although the vulnerability score was initially proposed for assessing fish sensitivity to fishing pressure^52^, it is grounded in the general assumption that large-bodied species with slow growth and late maturation are more inclined to be affected by disturbances^75,76^. Coherently, it discriminates vulnerable species depending on their life history attributes whereas the IUCN classification identifies species currently threatened on the basis of information on population dynamic, distribution range, and expert review^77^.

Regardless of the type of aquatic ecosystem, we found non-significant relationships between species richness and functional redundancies for the worst-case scenarios. This result demonstrates that even if the functional compensatory potential tends to increase with species richness (in lakes and rivers), some combinations of functional traits are supported by non-redundant species, so that their early loss induces larger impact on functional richness. For the three plausible scenarios (abundance-, trait- and IUCN-based scenarios), the functional alterations of lake assemblages were not significantly different from random expectation. On the contrary, the impacts of the three scenarios were greater than random species loss in river assemblages. A similar result was observed on estuarine assemblages for the abundance-based scenario. This scenario highlighted significant functional alterations when non-abundant species were removed early from river and estuarine assemblages, indicating that rare species support singular ecological functions, which were not shared by dominant species. The standardized effect sizes were particularly marked for estuaries, likely due to the structure of fish assemblages. They are generally composed of a few abundant species associated with a large number of rare species that occur in low number but have different habitat requirements^78^. In the same way, Mouillot, *et al*.^15^ showed that rare species among assemblages of coral reef fishes frequently support unique functions and significantly increase the level of functional diversity. The extinction of rare species can thus produce an important alteration of ecosystem functioning, since they promote different functions and ecosystem services, which in turn sustains local ecosystem properties^79^. Both trait- and IUCN-based scenarios revealed the same tendency, stressing that functional richness of river assemblages is more impacted by the loss of vulnerable species than for fish assemblages in lakes and estuaries. This high sensitivity is related to the low redundancy of riverine assemblages, which is concomitant with a large proportion of species listed as threatened in the IUCN Red List (on average 24.3% within assemblages) in comparison with lake and estuarine systems (respectively 7.7 and 5.1% on average). This result confirms conclusions of Toussaint, *et al*.^8^ which demonstrated that the extinction of IUCN threatened species is causing a sharp decrease in functional richness of freshwater fish community across the Eurasian realm. However, contrary to these results obtained at continental scale, our results indicate that the impact was significantly higher than expected. As a result, the spatial scale appears to be an important element to consider in determining weakly buffered sensitive areas against species extinction. Here, the local approach helps to identify and prioritize communities where conservation efforts should be planned to avoid the loss of functional diversity.

Our results highlighted differences in some functional indices between the two biogeographical regions, separated by the Pyrenees Mountains. Species and functional richness were generally higher in the Danubian region for the three aquatic ecosystems types, while there was little redundancy in the Iberian region for lakes and rivers. This suggests that Iberian Peninsula represent an area of high functional sensitivity for freshwater systems, possibly due to the high level of endemic species^27^ and taxonomic turnover of this region^80^. In this region, the redundancy of fish assemblages is very limited and the extinction of vulnerable species is expected to yield great functional alteration, according the trait- and IUCN-based scenarios. Similarly, the abundance-based scenario suggested that estuaries located in the Danubian region, and especially in the north-western France, are more sensitive to the loss of non-abundant species than others estuarine systems. Such identification of sensitive areas and communities is crucial to guide conservation efforts and could be used as an indicator in adaptive management approach to assess benefits of restauration actions^81^. However, given the variability in redundancy and sensitivity of fish assemblages within each aquatic ecosystem, it is undisputable that further investigations are required to determine the role of natural and anthropogenic factors. Indeed, the functional structure of assemblages is influenced by the specificity of local environmental conditions^38,41,82^ and can be impacted by (multiple) human stressors or the introduction of species^83,84^. For instance, Comte, *et al*.^64^ demonstrated that the presence of non-native species alter the biotic interactions between co-existing species, but leads to higher level of redundancy in communities. In such cases, non-native species can potentially play an important role to buffer the impact of species loss through their contribution to ecosystem process^85^.

To conclude, our assessment of functional redundancy based on species extinction scenarios allowed revealing specific patterns of functional sensitivity in fish assemblages of rivers, lakes and estuaries. Such approach provides quantitative criteria to evaluate the potential impact of species loss on ecosystem functioning, which are useful to determine conservation priorities. As it has already been reported, species richness alone is not sufficient to assess the functional vulnerability^9,40^. Our results suggest that the conservation effort should focus on freshwater systems and communities, particularly in the Iberian region, where the loss of threatened species will result in a marked decline of functional richness. In addition, the highly diverse functional traits of estuarine species should also deserve the attention of managers as rare species play a key functional role in this ecosystem. Nevertheless, our extinction scenarios were based on general assumptions on species’ sensitivity to environmental disturbance (abundance, traits, IUCN status) but do not take into account the species-specific response^85^. As a result, further research should examine the tolerance of species to the specific threats that act in the local systems to improve the accuracy of functional sensitivity assessment.

## Electronic supplementary material

Supplementary Information

Dataset
