# Supplementary material for: Functional redundancy and sensitivity of fish assemblages in European rivers, lakes and estuarine ecosystems
Source: Sci Rep. 2017 Dec 14;7:17611. doi: 10.1038/s41598-017-17975-x (PMC5730594; doi:10.1038/s41598-017-17975-x)
Supplement: Supplementary file 1 — Supplementary Information [file 41598_2017_17975_MOESM1_ESM.pdf]

Supporting Information

Functional redundancy and sensitivity of fish assemblages in European rivers, lakes and estuarine ecosystems

Nils Teichert, Mario Lepage, Alban Sagouis, Angel Borja, Guillem Chust, Maria Teresa Ferreira, Stéphanie Pasquaud, Rafaela Schinegger, Pedro Segurado and Christine Argillier

Relevance and description of functional traits used to describe fish species

Fish have numerous implications in ecosystem functioning because of their implication in food web dynamics, nutrient flows, or redistribution of bottom sediment (Holmlund and Hammer 1999). They are mobile fauna and have a major impact on the distribution and abundance of other organisms through trophic and competitive interactions. Accordingly, functional niches of fish were described based on five complementary traits, focusing on key elements determining species habitat preference and their position in the food web, i.e. fish size, vertical position, spawning habitat, trophic group, and swimming mode. These traits are commonly used to reflect the ecological functions of species in aquatic ecosystems (e.g. Eros et al. 2009, Guillemot et al. 2011, Mouillot et al. 2014, Parravicini et al. 2014, Pool et al. 2014).

- *<u>Body size</u> (six categories)*

Body size is a primary component of fish ecological niches (Wilson 1975). It is highly related to food-web structure, trophic levels, and energy flow in aquatic ecosystems (Emmerson and Raffaelli 2004, Jennings et al. 2001). Among other implications, the size of fish constrains the prey size, so that larger fish increase their minimum and maximum prey size (Costa 2009). Moreover, the body size is closely related to the demographic performances of species and their tolerance to environmental stress (Logez and Pont 2011, Winemiller and Rose 1992).

Fish size corresponds to the maximum total length reported in literature and was coded using six ordered categories: 0-8, 8.1-15, 15.1-30, 30.1-50, 50.1-80, and >80.1 cm.

- *<u>Position in the water column</u> (two categories)*

Position in the water column influence species interactions and the set of available prey, as well as the bentho-pelagic energy flow (e.g. Bellwood et al. 2006, Vander Zanden and Vadeboncoeur 2002). It

influences the species abundance along to the water column depending on availability and quality of habitats (e.g. sediment type, hydrodynamic condition, turbidity).

Position in the water column was expressed in two categories, i.e. benthic and non-benthic.

- *Spawning habitat (six categories)*

Spawning habitat is related to redistribution of bottom sediment, competition for ground habitats and mobility of early stages (Holmlund and Hammer 1999). It reflects specific environmental requirements for species reproduction purpose (e.g. sediment type, vegetation) that can be impacted by stream-bed alteration.

Spawning habitat was coded using six categories: lithophilic, pelagophilic, phytophilic, polyphilic, nest builder, internal brooder.

- *Diet (seven categories)*

Diet reflects the position in the food web and drives the trophic interactions with other ecosystem components (Power 1990, Vander Zanden et al. 1999). It affects the species repartition and habitat used depending on the availability of local resources (Seitz et al. 2014).

Species were assigned in seven trophic categories according to the dominant food item in the diet: piscivorous, omnivorous, planktivorous, insectivorous, herbivorous, detritivorous, and parasitic.

- *Swimming mode (eight categories)*

Swimming mode is defined by body-shape, swimming factor, and propulsion characteristic of fish, which describe locomotor performances in term of swimming speed and endurance and manoeuvrability (Helfman et al. 2009, Lindsey 1979). Locomotion behavior influences fish mobility, food acquisition and ability to escape from predation (Sibbing and Nagelkerke 2000). It is also closely related to species habitat requirements, e.g. depth, hydrodynamic, habitat configuration (Dumay et al. 2004), so that fish inhabiting complex environments have specialized swimming types (Helfman et al. 2009).

Swimming mode was coded using eight categories: carangiform, sub-carangiform, diodontiform, anguilliform, labriform, balistiform, amiiform, and rajiform.

References

Bellwood, D. et al. 2006. Functional versatility supports coral reef biodiversity. — Proceedings of the Royal Society of London B: Biological Sciences 273: 101-107.

Costa, G. C. 2009. Predator size, prey size, and dietary niche breadth relationships in marine predators. — Ecology 90: 2014-2019.

Dumay, O. et al. 2004. Functional groups of lagoon fish species in Languedoc Roussillon, southern France. — J Fish Biol 64: 970-983.

Emmerson, M. C. and Raffaelli, D. 2004. Predator–prey body size, interaction strength and the stability of a real food web. — J Anim Ecol 73: 399-409.

Eros, T. et al. 2009. Characterising functional trait diversity and trait-environment relationships in fish assemblages of boreal lakes. — Freshwater Biol 54: 1788-1803.

Guillemot, N. et al. 2011. Functional redundancy patterns reveal non-random assembly rules in a species-rich marine assemblage. — Plos One 6: e26735.

Helfman, G. et al. 2009. The diversity of fishes: biology, evolution, and ecology. — John Wiley & Sons.

Holmlund, C. M. and Hammer, M. 1999. Ecosystem services generated by fish populations. — Ecological Economics 29: 253-268.

Jennings, S. et al. 2001. Weak cross-species relationships between body size and trophic level belie powerful size-based trophic structuring in fish communities. — J Anim Ecol 70: 934-944.

Lindsey, C. 1979. Form, Function, and Locomotory Habits in Fish. — Fish physiology 7: 1-100.

Logez, M. and Pont, D. 2011. Development of metrics based on fish body size and species traits to assess European coldwater streams. — Ecol Indic 11: 1204-1215.

Mouillot, D. et al. 2014. Functional over-redundancy and high functional vulnerability in global fish faunas on tropical reefs. — P Natl Acad Sci USA 111: 13757-13762.

Parravicini, V. et al. 2014. Global mismatch between species richness and vulnerability of reef fish assemblages. — Ecol Lett 17: 1101-1110.

Pool, T. K. et al. 2014. Species contribute differently to the taxonomic, functional, and phylogenetic alpha and beta diversity of freshwater fish communities. — Divers Distrib 20: 1235-1244.

Power, M. E. 1990. Effects of fish in river food webs. — Science(Washington) 250: 811-814.

Seitz, R. D. et al. 2014. Ecological value of coastal habitats for commercially and ecologically important species. — ICES J Mar Sci 71: 648-665.

Sibbing, F. A. and Nagelkerke, L. A. 2000. Resource partitioning by Lake Tana barbs predicted from fish morphometrics and prey characteristics. — Rev Fish Biol Fisher 10: 393-437.

Vander Zanden, M. J. et al. 1999. Patterns of food chain length in lakes: a stable isotope study. — The American Naturalist 154: 406-416.

Vander Zanden, M. J. and Vadeboncoeur, Y. 2002. Fishes as integrators of benthic and pelagic food webs in lakes. — Ecology 83: 2152-2161.

Wilson, D. S. 1975. The adequacy of body size as a niche difference. — The American Naturalist 109: 769-784.

Winemiller, K. O. and Rose, K. A. 1992. Patterns of life-history diversification in North-American fishes - implications for population regulation. — Can J Fish Aquat Sci 49: 2196-2218.

Table S1: Mean values of the standardised effect size of area under the curve (SES AUC) calculated from the abundance-based, the trait-based and the IUCN-based scenarios for the two biogeographical regions covered by the study area, i.e. Danubian region and Iberian region. *P*-values indicate the significance of changes between regions for each aquatic system using Kruskal–Wallis non-parametric tests. Values in bold indicate *P*-value < 0.05.

	Lakes			Rivers			Estuaries		
	Danubian	Iberian	*P*-value	Danubian	Iberian	*P*-value	Danubian	Iberian	*P*-value
Abundance-based scenario	0.47	0.00	**0.003**	-0.46	-0.35	0.072	-1.87	-0.35	**< 0.001**
Trait-based scenario	0.71	0.33	**0.006**	-0.16	-0.43	**< 0.001**	0.01	0.69	**0.042**
IUCN-based scenario	0.21	0.06	0.656	-0.16	-0.47	**< 0.001**	-0.08	-0.16	0.935

Table S2: Pairwise comparisons between categories (small and large; headwater streams - HWS, medium gradient rivers - MGR, lowland rivers - LLR, and Mediterranean streams - MES) within the three aquatic systems, i.e. lakes, rivers and estuaries. The *P*-values of the post-hoc Kruskal-Wallis tests are indicated for the abundance-based scenario (upper diagonal matrix) and the trait-based scenario (lower diagonal matrix). Values in bold indicate *P*-value < 0.05.

		Lakes		Rivers				Estuaries	
		small	large	HWS	MGR	LLR	MES	small	large
Lakes	small		0.845	-	-	-	-	-	-
	large	0.907		-	-	-	-	-	-
Rivers	HWS	-	-		0.16	0.58	0.999	-	-
	MGR	-	-	0.999		0.999	0.86	-	-
	LLR	-	-	**0.001**	**< 0.001**		0.999	-	-
	MES	-	-	**< 0.001**	**< 0.001**	0.999		-	-
Estuaries	small	-	-	-	-	-	-		0.609
	large	-	-	-	-	-	-	0.592	

Table S3: Pairwise comparisons between categories (small and large; headwater streams - HWS, medium gradient rivers - MGR, lowland rivers - LLR, and Mediterranean streams - MES) within the three aquatic systems, i.e. lakes, rivers and estuaries. The *P*-values of the post-hoc Kruskal-Wallis tests are indicated for the IUCN-based scenario. Values in bold indicate *P*-value < 0.05.

		Lakes		Rivers				Estuaries	
		small	large	HWS	MGR	LLR	MES	small	large
Lakes	small		0.658	-	-	-	-	-	-
	large			-	-	-	-	-	-
Rivers	HWS				**0.001**	0.999	0.999	-	-
	MGR					0.997	**0.005**	-	-
	LLR						0.999	-	-
	MES							-	-
Estuaries	small								0.499
	large								
